# Feasibility of co-polarizing ¹³C-pyruvate and ¹³C-*tert*-butanol for simultaneous metabolic and perfusion imaging

**DOI:** 10.1038/s44303-026-00172-9

**Published:** 2026-05-18

**Authors:** Karen Dos Santos, Samuel R Calos, Qianhui Dou, Atsushi M. Takahashi, Leo L. Tsai, Aaron K. Grant, Yi-Fen Yen

**Affiliations:** 1 Athinoula A. Martinos Center for Biomedical Imaging, Massachusetts General Hospital, Harvard Medical School, Charlestown, MA USA; 2 Polarize ApS., Asmussens Alle 1, 1808 Frederiksberg, Denmark; 3 McGovern Institute for Brain Research, Department of Brain and Cognitive Sciences, Massachusetts Institute of Technology, Cambridge, MA USA; 4 Division of Abdominal Imaging, Department of Radiology, Massachusetts General Hospital, Harvard Medical School, Boston, MA USA; 5 Department of Radiology, Beth Israel Deaconess Medical Center, Harvard Medical School, Boston, MA USA

**Keywords:** Biochemistry, Biological techniques, Chemistry

## Abstract

Altered metabolism and perfusion are hallmarks of numerous diseases, yet simultaneous evaluations in vivo remain limited. We present a dual-purpose hyperpolarized probe combining [1-¹³C]pyruvate and ¹³C-*tert*-butyl alcohol (^13^C-TBA) for concurrent metabolic and perfusion imaging. An optimized formulation produced high polarizations with short build-up times. In vivo imaging in healthy and tumor-bearing rats demonstrated robust signals of TBA and pyruvate metabolism. This co-polarized dual probe offers strong preclinical and translational potential.

Hyperpolarized (HP) magnetic resonance imaging (MRI) and MR spectroscopic imaging (MRSI) have emerged as a powerful modality for real-time and noninvasive assessment of tissue metabolism^1^. Dissolution-Dynamic Nuclear Polarization (d-DNP) enabled the detection of [1-^13^C]pyruvate metabolism in animals^2^, and in humans^3^, marking a major breakthrough in MR-based metabolic imaging. While HP [1-^13^C]pyruvate provides rich metabolic information, combining it with perfusion imaging offers an even more powerful tool for characterizing disease. Perfusion governs the delivery of nutrients and oxygen, playing a critical role in disease onset and progression, which in turn sets metabolic constraints^4^. The gold standard for contrast-based perfusion MRI relies on the injection of gadolinium-based contrast agents (GBCAs). However, administration of GBCAs carries known risks, and cumulative gadolinium deposition in bone, brain, and kidneys in patients undergoing multiple examinations is also a potential concern^5–7^. Hyperpolarized agents may provide an alternative that avoids gadolinium toxicity. To address perfusion imaging, several HP ^13^C-labeled tracers such as HP001^8^, urea^9^, and others^10,11^ have been proposed, including a proof-of-concept in humans with urea and pyruvate^12–15^. Although these tracers offer high sensitivity - and in the case of [¹³C,¹⁵N₂]-urea, notably long T₂ relaxation times^9^ - their limited permeability into tissues poses a challenge for detecting microvascular or interstitial abnormalities.

[2-^13^C]*tert*-butyl alcohol (^13^C-TBA) offers distinct advantages due to its ability to freely diffuse across biological membranes, including the blood-brain barrier, as we and others previously reported^16,17^. As a result, TBA shows a high signal in tissue (as opposed to vessels) and has similar properties to the PET tracers H_2_^15^O and ^11^C butanol, which are considered the non-invasive gold standard for perfusion quantitation^18–21^. We recently showed that hyperpolarized TBA provides high sensitivity and long tissue residence time to support three-dimensional quantitative mapping of T_1_ and T_2_ relaxation times and cerebral blood flow with isotropic resolutions of 1.2 - 1.5 mm³ in the rat brain, even under ischemic conditions, and a high resolution of 0.48 mm³ isotropic in qualitative perfusion-weighted imaging of rat cortical gray matter^22^.

In this study, we developed and optimized a co-polarization strategy for [1-¹³C]pyruvate and ^13^C-TBA as a dual-purpose probe for simultaneous metabolic and perfusion imaging. In this approach, ¹³C-pyruvate uniquely serves three roles: as a glassing agent, solvent for TBA, and a hyperpolarized metabolic probe.

First, natural abundance pyruvate and TBA were combined in varying volume ratios, from 10%/90% to 90%/10%, to evaluate their miscibility and capacity to form a homogeneous glassy matrix upon freezing in liquid nitrogen. All mixtures were fully miscible and appeared visually glassy upon freezing. Our final 60 μL sample formulation consisted of 30% pyruvate and 70% ^13^C-TBA v/v. This sample preparation yields, after dissolution, a final concentration of 80 mM [1-^13^C]pyruvate and 135 mM ^13^C-TBA, similar to the concentration previously achievable individually as a single hyperpolarized probe^22,23^.

We then evaluated the effect of radical type and concentration on pyruvate polarization levels in natural abundance TBA (Fig. 1). All liquid-state polarization levels and solid-state build-up times are provided in Table S1. As shown in Fig. 1A, both trityl AH111501 (herein, AH) and trityl OX063 (herein, OX) yielded increasing polarization up to 25 mM, beyond which polarization values decreased. Trityl AH consistently produced higher polarization percentages compared to trityl OX, with peak values approaching 47% at 21-25 mM. The polarization buildup time, quantified by the solid-state polarization time constant (*T*_*p*_), was also dependent on radical type. *T*_*p*_ refers to the polarization build-up time constant of the co-polarized sample, as measured from the total ¹³C signal of the mixture during the solid-state DNP build-up. It therefore reflects the overall polarization dynamics of the co-polarized system rather than that of ¹³C pyruvate or ^13^C-TBA individually. Samples with OX exhibited shorter *T*_*p*_ values compared to trity AH at equivalent radical concentrations (Fig. 1B), indicating faster solid-state polarization kinetics, albeit yielding lower polarization levels (Fig. 1A).

**Fig. 1:**
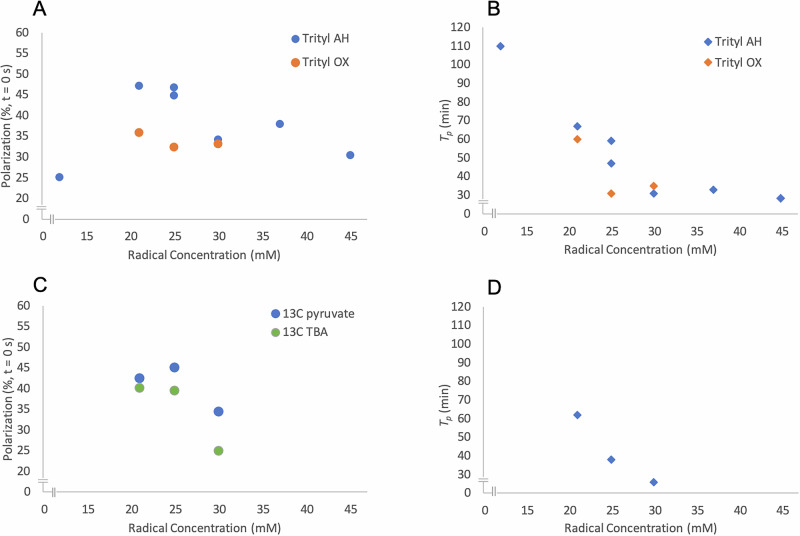
Hyperpolarization of [1-¹³C]pyruvate in a natural abundance TBA formulation and inpartially labelled^13^ C-TBA across a range of radical concentrations. **A** Liquid-state polarization (%) measured at 1.4 T and 27 °C and back-calculated to the moment of dissolution, *P*(*t* = 0 s). Data are shown for trityl AH (blue circles) and trityl OX (orange circles) across radical concentrations of 12, 21, 25, 30, 37, and 45 mM. Trityl AH produced consistently higher polarization than trityl OX, with peak values observed near 21–25 mM. **B** Solid-state polarization buildup time constants (*T*_*p*_) for the same radical concentrations. Trityl AH (blue diamond) exhibited longer *T*_*p*_ values compared to trityl OX (orange diamond), indicating slower polarization kinetics. These results support the use of trityl AH near 25 mM as an optimal condition for efficient polarization. **C** Liquid-state polarization (%) using trityl AH for [1-^13^C]pyruvate (blue circle) and ^13^C-TBA (green circles) using a 1:1 (v/v) ratio between ^13^C- and natural abundance TBA. **D** *T*_*p*_ of the co-polarization buildup across 21, 25, and 30 mM AH concentrations further confirms the use of trityl AH at 25 mM. For improved visualization, the origin point has been omitted in each plot.

It is noteworthy that all samples were polarized using a relatively low microwave power of 2.5 mW, which, based on our previous experience, provides the optimal performance in our polarizer system. Faster polarization buildup times may be achievable with higher microwave power, although final polarization levels may vary.

Second, we selected the three best-performing concentrations of trityl AH (21, 25, and 30 mM) and prepared co-polarized samples containing [1-^13^C]pyruvate and ^13^C-TBA. To be mindful of the cost of ^13^C-TBA, we used a 1:1 (v/v) mixture of ¹³C-TBA and natural abundance TBA for these measurements. Since the DNP polarization build-up depends on the spatial distribution of radicals in the solution and their proximity to the nuclei being polarized, we expect this sample to exhibit behavior comparable to that of fully isotopically labeled ^13^C-TBA. Protonated ^13^C-TBA was employed in all studies reported here, although deuterated ^13^C-TBA^22^ would likely present longer relaxation times. Both metabolic probes achieved substantial polarization across all conditions (Fig. 1C, D). At 21 and 25 mM, polarization levels were comparable for both molecules (around 40%); however, the polarization buildup time was significantly shorter at 25 mM (38 min, instead of 62 min). With an even higher trityl AH concentration at 30 mM Trityl AH, the polarization buildup time was shortened further (*T*_*p*_ = 26 min), but the degree of polarization also decreased for both molecules (pyruvate = 34%, TBA = 25%).

We acknowledge that the literature reports 30 mM as optimal for [1-^13^C]pyruvate at 6.7 T^24,25^, which is slightly higher than what we observed using our co-polarized sample formulation. Further studies are necessary to assess whether gadolinium doping, which shortens the trityl electron spin-lattice relaxation time and has a strong effect in boosting ^13^C spin polarization, could potentially increase liquid-state polarization levels^25^.

Third, we selected trityl AH at 25 mM as the optimal balance between high polarization levels and a short polarization buildup time. The final formulation containing [1-¹³C]pyruvate and fully enriched ¹³C-TBA yielded 43.6% for pyruvate and 44.4% for ¹³C-TBA (T₁ = 71 s and 33 s, respectively, at 1.4 T), both suitable for in vivo imaging.

Finally, to demonstrate the feasibility of this hyperpolarized probe in vivo, we performed imaging studies on the rat kidney, liver, liver tumor, and brain following intravenous injections of co-polarized [1-¹³C]pyruvate and ^13^C-TBA (Figs. 2–4). Because of the wide chemical shift separation (>90 ppm) between ^13^C-TBA and [1-¹³C]pyruvate metabolites, we first acquired Chemical Shift Imaging (CSI) to illustrate the spectral and spatial distribution of all metabolite and ^13^C-TBA signals in healthy rat kidneys at 4.7 T by using a dual-tuned ^1^H/^13^C volume coil (Bruker, Billerica, MA, USA), resulting in well-resolved images of TBA, pyruvate, lactate, and alanine (not shown) (Fig. 2). A CSI spectrum summed over all voxels within a region of interest (ROI) encompassing both kidneys revealed a strong TBA signal and clear metabolic conversion of pyruvate to lactate, alanine, and bicarbonate (Fig. 2B). All compounds exhibit their strongest signal intensities in the kidneys and supplying vessels, as expected (Fig. 2A). The CSI acquisition was slow (24.1 s per image), and the signals vanished after the second frame. To illustrate the signal time course in vivo with a better temporal resolution, we acquired non-selective spectroscopic data repeated every 3 s over the kidney region in a second rat at 9.4 T, by using a 6 cm ^13^C surface loop transceiver coil placed underneath the rat abdomen. The signal time curves showed the arrival of the injected HP [1-¹³C]pyruvate and ^13^C-TBA, followed shortly by the metabolic production of lactate, alanine, and bicarbonate (Fig. 2C). At later time points, the [1-¹³C]pyruvate and ^13^C-TBA curves showed a smooth decay that was fitted with a mono-exponential decay curve (not shown), resulting in a decay time constant of 12.1 s for [1-¹³C]pyruvate and 21.4 s for ^13^C-TBA. The long-lasting ^13^C-TBA signal was expected, consistent with the prolonged interstitial retention due to ^13^C-TBA’s large distribution volume reported in our previous study^22^, using HP ^13^C-TBA alone.

**Fig. 2:**
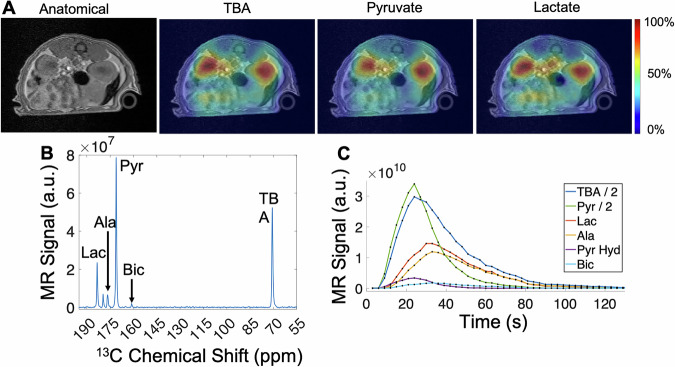
Spatial, spectral, and temporal distributions of co-polarized [1-^13^C]Pyruvate and ^13^C-TBA in healthy rat kidneys. **A** Chemical shift imaging (CSI) of TBA, pyruvate, and lactate maps (colors) acquired at 4.7 T are overlaid on T_1_-weighted proton anatomical images. **B** The ¹³C spectrum was summed over an ROI covering both kidneys from the first two time frames of the CSI data at 4.7 T. Due to the poor temporal resolution in CSI (22.5 s), the ^13^C signal vanished after the second frame. **C** To illustrate the signal dynamics with a better temporal resolution, non-selective dynamic spectroscopy data with 3 s resolution were acquired at 9.4 T by using a 6 cm ^13^ C surface coil placed underneath the kidney region of a second rat. ^13^C-TBA shows a long-lasting signal in vivo. The dynamic curves in (**C**) were not corrected for RF depletions. Images in (**A**) were not corrected for coil sensitivity. The colors of each metabolite map are normalized to the maximum signal within each map, where the maximum signal is displayed in red (100%).

**Fig. 3:**
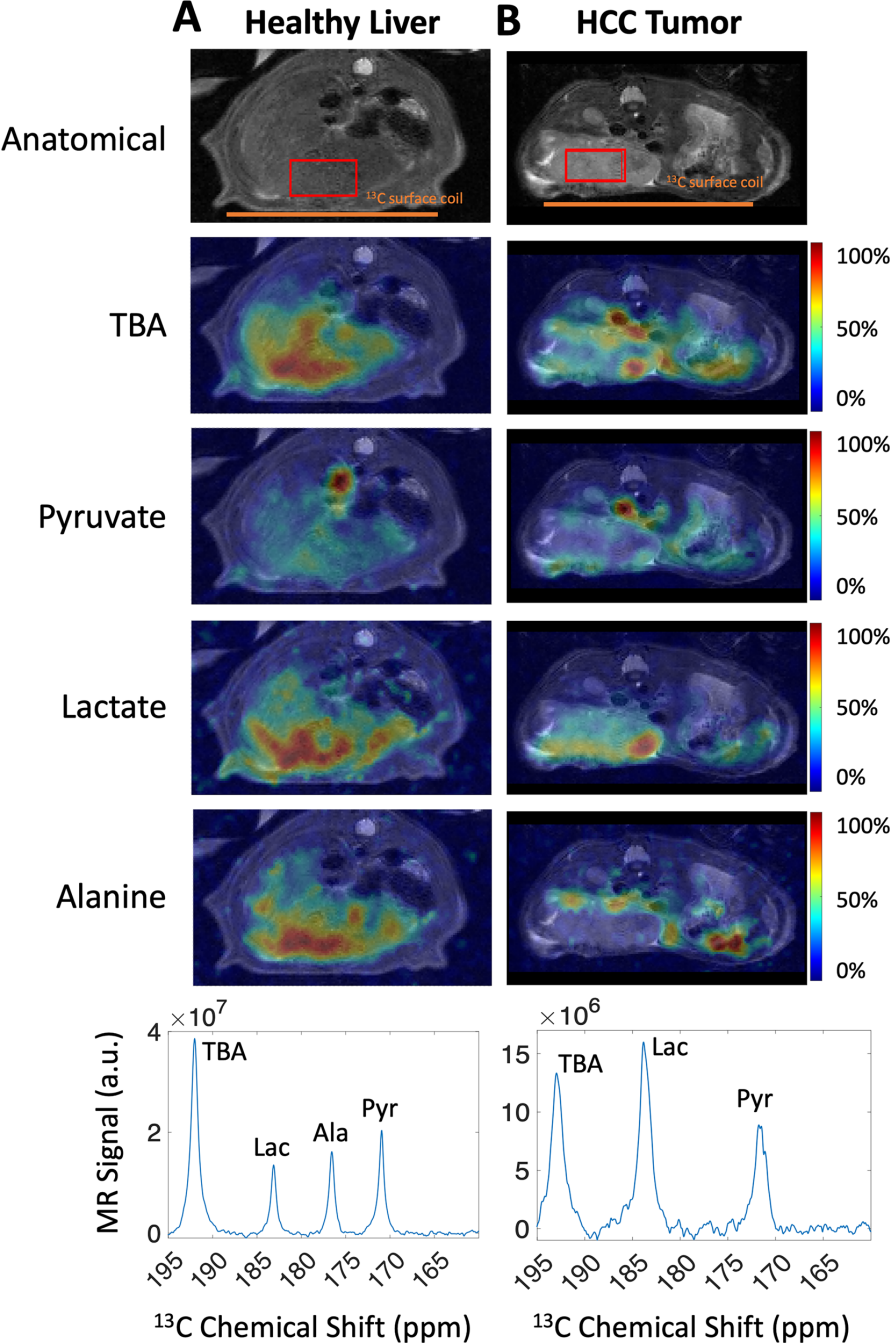
Time-resolved echo-planar spectroscopic imaging (EPSI) of co-polarized [1-¹³C]Pyruvate and ¹³C-TBA in rat liver acquired at 4.7 T. ^13^C images of TBA, pyruvate, lactate, and alanine (colors) are overlaid on T_2_-weighted proton anatomical images in a healthy (**A**) and tumor-bearing rat liver (B). In (B), a magnitude spectrum summed over the HCC tumor ROI (red box in (**B**)) shows a higher lactate signal relative to pyruvate than the ROI spectrum over the healthy liver in (**A**), with minimal alanine in HCC 12 days after implantation. Images were not corrected for coil sensitivity. The colors of each metabolite map are normalized to the maximum signal within each map, where the maximum signal is displayed in red (100%). The metabolite signal time curves from the ROIs are shown in the Supplementary Information (Fig. S1).

**Fig. 4:**
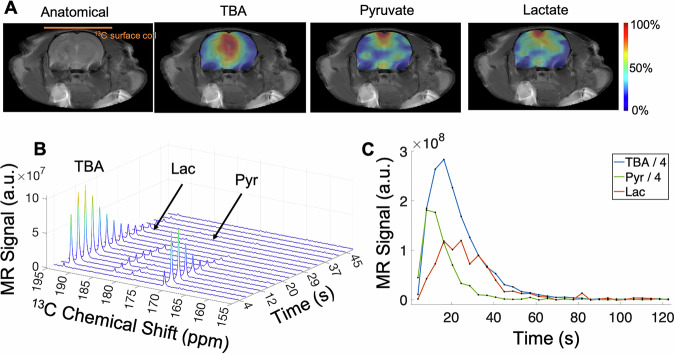
Time-resolved EPSI of co-polarized [1^-13^C]Pyruvate and ^13^C-TBA in healthy rat brain acquired at 9.4 T. **A** ¹³C images of TBA, pyruvate, and lactate (colors) are overlaid on T_2_-weighted proton anatomical images. **B** Temporal changes (stack-plot) of the spectra summed over the brain ROI show pyruvate metabolic productions and large TBA signals. **C** Dynamic curves highlight the prolonged TBA signal in the brain. The colors of each metabolite map are normalized to the maximum signal within each map, where the maximum signal is displayed in red (100%). Images were not corrected for coil sensitivity. The stack-plot and dynamic curves were not corrected for RF depletions.

For a more efficient MRSI method, we exploited Echo-Planar Spectroscopic Imaging (EPSI). Figure 3A presents EPSI of a rat with a healthy liver, and Fig. 3B a rat bearing a hepatocellular carcinoma (HCC) tumor, using a custom-built 6 cm ^13^C transceiver surface coil placed underneath the abdomen. While EPSI offers inherent efficiency, it is limited by a trade-off between spatial resolution and spectral bandwidth^26^. The wide chemical shift separation (>90 ppm) between the [1-¹³C]pyruvate/metabolite resonances and the distant ¹³C-TBA peak (Fig. 2B) would have presented a considerable challenge for simultaneous spectral coverage with a reasonable spatial resolution. To overcome this, a 40 ppm spectral bandwidth was intentionally configured to encompass the pyruvate, pyruvate-hydrate, alanine, and lactate peaks, while strategically aliasing the ¹³C-TBA peak to appear within the spectral bandwidth at 192 ppm without spectral overlap with any metabolic resonances (Fig. 3)^26,27^. This optimized approach enabled the simultaneous visualization of key signals within a constrained spectral window while achieving a reasonable 2.5 × 2.5 mm in-plane resolution with a temporal resolution of 4 s per slice. The spectroscopic images demonstrate the spatial distribution of metabolic and perfusion markers, with distinct pyruvate, alanine, lactate, and TBA signals in the ROI spectra. Clear differences emerged between the healthy liver (Fig. 3A) and HCC tumor (Fig. 3B), including largely reduced alanine and elevated lactate levels in the HCC tumor. The TBA signal in the tumor appears to be smaller than in the healthy liver. This is consistent with the N1S1 model, which at later stages is known to be necrotic with a low density of microvessels^28^, resulting in a hypoxic environment known to be a driver of glycolysis^29^.

The images shown in Fig. 3 highlight another difference between the tissue kinetics of TBA and pyruvate. These images were timed to coincide with the arrival of the intravenous contrast bolus in tissue, expected to be 5-10 s after the end of the injection^22^. Contrary to what might be expected, the images show a prominent pyruvate signal in the large vessels of the abdomen and a lower vascular signal from TBA. This difference in signal occurs because, after the co-polarized bolus has passed through the capillary bed the first time, freely diffusible TBA is largely extracted into tissue^17^, while pyruvate, which is partially permeable in most tissues, continues to have a high intravascular concentration. For this reason, pyruvate continues to show high intravascular signal for an extended period of time, similar to what is observed with gadolinium, while TBA generally does not show elevated signal in vessels. While time-of-flight effects can accentuate venous signal from TBA in 2D imaging^17^, TBA’s concentration in venous blood and recirculating arterial blood is generally expected to be comparatively modest.

Figure 4 shows time-resolved EPSI data of a healthy rat brain. The pyruvate signal is dominated by large vessels, most prominently on the top of the brain near the sagittal sinus vein. The lactate signal is also strong in the sagittal sinus vein but is well distributed in the cortex, hippocampus, and thalamus, indicating active metabolic conversion in the brain. ¹³C-TBA shows a stronger signal intensity medial in the brain, but an overall well-distributed signal, reflecting efficient tissue perfusion. Moreover, the spectral stack plot over time (Fig. 4B) and metabolite signal time curves (Fig. 4C) from the brain ROI highlight the long-lasting TBA signal in the brain and well-resolved chemical shifts.

These feasibility data provide important timing and signal-to-noise insights to inform future sequence design and optimization strategies for higher-resolution ¹³C-TBA mapping, similar to what we previously achieved using HP ¹³C-TBA alone^22^, without compromising the spatial resolution for pyruvate and its downstream metabolites. Additional studies are warranted to assess whether ¹³C-TBA alters pyruvate metabolism or elicits other physiological interactions. Future investigations should also incorporate tumor models spanning a range of microvessel densities and include comparisons against established perfusion imaging approaches, such as dynamic contrast-enhanced imaging with gadolinium or arterial spin labeling perfusion MRI.

While caution is clearly warranted with respect to the biocompatibility of ¹³C-TBA, its toxicity is expected to be low. The intravenous LD50 in mice is roughly 1500 mg/kg^30^, far above the ~100 mg/kg dose needed for perfusion imaging. The bioeffects, metabolism, and pharmacokinetics of TBA are well understood because it is a metabolic by-product of the fuel additive methyl *tert*-butyl ether (MTBE), which has been studied in rodents and humans. These studies^31,32^ show that TBA is metabolized to form the excretory metabolites *tert*-butanol-glucuronide, 2-methyl-1,2-propanediol, and 2-hydroxyisobutyrate. The half-life in blood is roughly 5–7 h. Prior studies in animals show that the doses necessary for imaging are well-tolerated and that the toxic effects are limited^33–35^. In the brain, TBA is expected to have intoxicating effects similar to ethanol^34,36^. The tracer washes out of the brain on a timescale of roughly 1 min and eventually equilibrates at a concentration of roughly 0.01-0.02% (v/v), a level below the 0.08% blood concentration of ethanol that is considered the legal limit for operating a motor vehicle in much of the US.

While it would clearly be desirable to quantify the in vivo *T*_*1*_ decay rates of [1-¹³C]pyruvate and ¹³C-TBA, this analysis is complicated by the tissue kinetics of these tracers. The signal decay of ¹³C-TBA in tissue depends on its *T*_*1,2*_ relaxation times and the imaging parameters (choice of sequence and tip angle) as well as local blood flow. Pyruvate signal trends have additional dependence on tissue permeability and active transport. Full analysis of these effects is left to future work. However, fits to the TBA signal in the rat brain suggest *T*_*1*_*≈* 40 s in cortical gray matter at 9.4 T in our previous work^22^.

Perfusion quantitation requires imaging with sufficient temporal resolution to fully characterize the bolus passage through tissue. In gadolinium-based dynamic susceptibility contrast (DSC) imaging, ~1 s resolution is required to capture the relatively fast passage of intravascular GBCAs through tissue^37^, particularly in the brain. Diffusible ¹³C-TBA generally has longer residence times, permitting the use of slower ~3-4 s resolution for imaging the signal in tissue. Our previous studies in the rat brain using hyperpolarized ^13^C-TBA alone^22^ have obtained quantitative perfusion maps at 2.8 s temporal resolution and 1.2 mm three-dimensional isotropic spatial resolution, albeit with higher temporal resolution for sampling of the arterial input. Similarly, perfusion quantitation with urea has been accomplished at 4 s temporal resolution and 2.6 × 2.6 mm spatial resolution^13^.

Compared to hyperpolarized ^13^C-urea as perfusion tracer, ^13^C-TBA offers distinct advantages, particularly its ability to freely diffuse across biological membranes, including the blood–brain barrier. Following bolus injection, ^13^C-TBA distributes with blood flow to the capillary bed, where it diffuses into the larger extravascular space and washes out slowly over tens of seconds (i.e., longer retention time). In contrast, urea and other non-diffusible tracers remain largely intravascular, passing rapidly through tissue. The slower washout of ^13^C-TBA permits extended three-dimensional imaging and yields a higher SNR, which permits higher spatial resolution images. In our previous work^22^, we demonstrated qualitative perfusion-weighted imaging at 480 µm resolution using hyperpolarized ^13^C-TBA alone using a balanced Steady-State Free Precession (bSSFP) sequence. It is technically more challenging to achieve a similar high resolution while imaging multiple metabolites simultaneously. However, we do anticipate extending our work and developing a new multi-echo acquisition technique in the near future to acquire brain images with this new dual-agent sample formulation at a high spatial resolution approaching what we have previously achieved by using hyperpolarized ^13^C-TBA alone.

This study presents, for the first time, a dual-purpose DNP probe combining [1-¹³C]pyruvate and ¹³C-TBA for simultaneous in vivo imaging of metabolism and perfusion. The formulation was optimized to achieve both the highest possible solid-state polarization and the shortest polarization buildup time in a d-DNP polarizer at 6.7 T, through comparative evaluations of trityl radicals AH111501 and OX063. Consistently high and reproducible liquid-state polarizations were achieved for both [1-¹³C]pyruvate and ¹³C-TBA. Previous work reported a liquid-state polarization of approximately 34% at the time of dissolution for ¹³C-TBA in a sucrose/water matrix^22^, whereas our co-polarized formulation achieves around 44% liquid-state polarization for both [1-¹³C]pyruvate and ¹³C-TBA. The results showed that TBA provided well-resolved perfusion images, while high-quality metabolic images of pyruvate and its downstream metabolites were obtained concurrently, exhibiting the expected metabolic profiles characteristic of the brain, kidney, liver, and the tumor model. This approach may have several advantages over the current approach of ^13^C metabolic imaging followed by ^1^H perfusion imaging with gadolinium contrast: single-injection protocol, simultaneous acquisition of metabolic and perfusion data, decreased total acquisition time, and avoidance of gadolinium toxicity, although high-resolution metabolite and perfusion maps with this co-polarized dual agent sample and its feasibility to translate for clinical research are yet to be demonstrated.

Future research will expand acquisition methodologies, including rapid multi-echo imaging sequences to derive additional parameters (e.g., diffusion and relaxation times), and deepen insights into perfusion–metabolism relationships. The co-polarized probe holds significant potential in diseases marked by metabolic and microvascular changes, including cancer, neurological disorders, and myocardial diseases. Collectively, these advancements aim to establish hyperpolarized ¹³C MRI towards a robust multi-probe imaging platform for preclinical and clinical applications.

## Methods

### Sample optimization

Natural abundance pyruvate and TBA (Sigma-Aldrich, USA) were mixed in varying volume ratios ranging from 10%/90% to 90%/10% to assess miscibility and the ability to form a homogeneous glassy matrix upon freezing in liquid nitrogen. All mixtures were miscible and appeared glassy upon freezing. The final 60 μL formulation consisted of 30% pyruvate and 70% TBA v/v.

To optimize radical concentration, [1-^13^C]pyruvate (Sigma-Aldrich, USA) and natural abundance TBA formulation were hyperpolarized using a SpinAligner Polarizer (Polarize ApS, Denmark) operating at 6.7 T and 1.3 K. To monitor the build-up of solid-state DNP-enhanced polarization in real time, 0.4° low-flip-angle excitations and 4 signal averages were used. The corresponding signal intensities were integrated and displayed in real-time on the polarizer, accompanied by an exponential fit representing the polarization kinetics and providing the solid-state buildup time *T*_*p*_^38^.

A range of trityl radical concentrations was evaluated, with Trityl AH (AH111501, Polarize ApS, Denmark) at concentrations of 12 mM, 21 mM, 25 mM, 30 mM, 37 mM, and 45 mM, while Trityl OX (OX063, Polarize ApS, Denmark) was tested at 21 mM, 25 mM, and 30 mM. All samples were polarized until the polarization reached a plateau, thereby ensuring that steady-state polarization was achieved in each case.

Polarization measurements were confirmed with [1-^13^C]pyruvate and ^13^C-TBA (Sigma-Aldrich, USA), 1:1 v/v with natural abundance TBA for Trityl AH at concentrations of 21, 25, and 30 mM. For animal experiments, a sample containing [1-^13^C]pyruvate, ^13^C-TBA, and 25 mM of Trityl AH was prepared.

All samples were hyperpolarized until maximum polarization and subsequently dissolved with 3.4 mL of dissolution buffer containing 40 mM Trizma PreSet Crystals pH 7.6, 50 mM sodium chloride, 0.27 mM disodium ethylenediaminetetraacetate dihydrate (EDTA), and 50 mM sodium hydroxide. After dissolution, the final solution of 80 mM [1-^13^C]pyruvate and 135 mM ^13^C-TBA, with pH 7, was collected and transferred either (i) to the scanner for animal experiment in approximately 30 s inside a hand magnet of 1 T or (ii) directly to the benchtop spectrometer in 15.2+/− 0.5 s (*n* = 8) for polarization measurements.

### Liquid-state polarization measurements

Following dissolution, the resulting liquid-state polarization and *T*_*1*_ relaxation were measured at 1.4 T Spinsolve 60 MHz Multi-X PLUS spectrometer (Magritek Inc., PA, USA) using a 10 mm broadband NMR probe at 27 °C. After dissolution, approximately 600 ${\rm{\mu }}$L of the hyperpolarized sample was inserted into a 5 mm NMR tube. The time delay from dissolution to the start of the dynamic spectroscopy acquisition on the benchtop spectrometer was approximately 15 s. Non-selective ^13^C spectra using a hard pulse were acquired using 160 ppm center frequency, ^1^H-^13^C decoupling, 5^o^ flip angle, with a temporal resolution of 2 s for over 2 min. Thermal equilibrium (TE) spectra were acquired after addition of 15 μL of clinical Dotarem (gadoterate meglumine, 0.5 mmol/mL, Guerbet, USA) per mL of ^13^C solution, using 90 ^o^ flip angle, with repetition time of 10 s, for over 45 min. The data were processed with MestReNova software (MestReLab Research, Santiago de Compostela, Spain). FIDs were zero-filled to 16384, apodized with a 1 Hz exponential function, phased, and baseline corrected. After integration, the signal time curve was fitted to a monoexponential decay function to determine the observed *T*_*1*_ constant (*T*_*1*_^obs^), Eq. 1, and corrected for the RF depletion to estimate *T*_*1*_, Eq. 2:

1 $${S}^{{obs}}\left(t\right)=\,{S}_{0}\cdot {e}^{-\,\frac{t}{{T}_{1}^{{obs}}}}$$

Where S_0_ is the initial signal, *T*_*1*_^obs^ is the observed relaxation time. To obtain the longitudinal relaxation time *T*_*1*_, *T*_*1*_^obs^ was corrected by the flip angle α^TE^:

2 $${T}_{1}={{T}_{1}^{{obs}}\{1+\frac{{T}_{1}^{{obs}}}{{TR}}\mathrm{ln}[\cos ({\alpha }^{{TE}})]\}}^{-1}$$

Polarization levels in liquid-state were calculated using Eq. 3:

3 $$P={P}_{{TE}}\cdot \varepsilon =\frac{{S}^{{HP}}}{{S}^{{TE}}}\cdot \frac{{{NS}}_{{acq}}^{{TE}}}{{{NS}}_{{acq}}^{{HP}}}\cdot \frac{\sin \left({\alpha }^{{TE}}\right)}{\sin \left({\alpha }^{{HP}}\right)}\cdot \frac{{{RG}}^{{TE}}}{{{RG}}^{{HP}}}$$

where *P*_*TE*_ is the polarization in thermal equilibrium, *S*^x^ is the integral of the respective signal, NS^x^_acq_ is the number of accumulated spectra, α^x^ is the excitation flip angle, and RG^x^ is the linear receiver gain for hyperpolarized (*x* = HP) and thermally polarized (*x* = TE) NMR^38^.

### Animal experiment

All animal experimental procedures were performed in accordance with the protocol number 2019N000126 approved by the Institutional Animal Care and Use Committee (IACUC) at Massachusetts General Hospital (MGH) and followed the guidelines of the Institute of Laboratory Animal Resources, National Research Council. Rats were housed in a temperature-controlled environment maintained at 22 ± 1 °C with a 12-h light/dark cycle. Their general well-being was assessed daily to detect any signs of distress or abnormal behavior.

For animal imaging, anesthesia was induced initially with 3–4% isoflurane in 0.5–1.0 L/min oxygen and maintained at 1–2.5% isoflurane in 1.0–1.5 L/min of enriched air (approximately 30% O_2_) throughout the experiment. For injection, the lateral tail vein was cannulated with a 23-gauge needle connected to a 100-cm polyethylene extension tubing with a 0.58 mm inner diameter (PE50, Braintree Scientific Inc., MA, USA). Animals were positioned prone on a custom-designed, 3D-printed MRI-compatible cradle. In studies that employed a surface coil, the coil was centered over the anatomical region of interest to optimize surface coil sensitivity. Body temperature was monitored by using a rectal thermometer and maintained at 36–37 °C by a circulating warm water blanket, and respiratory rate and temperature were continuously monitored (SA Instruments, USA). During imaging, animals received 10 μL/g body weight of HP [1-^13^C]pyruvate and ¹³C-TBA solution over about 15 s. All animals recovered successfully after imaging. This animal study was conducted as an IACUC-approved survival study; therefore, animals were not euthanized at the completion of the study.

### Cell line and HCC model

The rat hepatocellular carcinoma cell line N1S1 (ATCC, CRL-1604™, male origin) was sourced from the American Type Culture Collection (Manassas, VA). Cells were cultured at 37 °C in a humidified incubator with 5% CO₂, using Dulbecco’s Modified Eagle’s Medium (GIBCO) supplemented with 1% antibiotic-antimycotic solution (100x) (GIBCO). A female Fischer (CDF) rat (Charles River Laboratories, Germantown, MD), weighing around 130 g, was utilized for this study. Tumor implantation was achieved through the injection of 1 × 10⁶ N1S1 cells, suspended in 50 μL PBS, into the left hepatic lobe under aseptic conditions. Hyperpolarized ¹³C MRI was conducted 12 days after implantation.

### MRI hardware

The majority of the animal imaging was performed on a 4.7 T MRI scanner (Bruker BioSpec USR 47/40, Bruker, Billerica, MA, USA) using a 12 cm gradient insert with a maximum field strength of 66 G/cm and ParaVision 360 imaging software. For the EPSI experiment of the healthy liver and liver tumor model, a Bruker ^1^H quadrature birdcage coil (86 mm inner diameter) was used for ^1^H anatomical imaging, and a custom-built ^13^C transceiver surface coil (6 cm in diameter) was used for HP ^13^C MRI. For the CSI experiment, a Bruker ^1^H/^13^C linear birdcage coil (72 mm inner diameter) was used for both ^1^H and ^13^C acquisitions.

For the acquisition of non-localized dynamic spectroscopy in kidneys and the EPSI experiment in the brain, data acquisition was performed on a 9.4 T MRI scanner (Bruker BioSpec USR 47/40, Bruker, Billerica, MA, USA) using a 12 cm gradient insert with a maximum field strength of 66 G/cm and ParaVision 360 imaging software. A Bruker ^1^H quadrature birdcage coil (86 mm inner diameter) was used at 9.4 T for ^1^H anatomical imaging. A custom-made ^13^C transceiver surface coil (6 cm in diameter) placed dorsal to the kidneys was used to collect dynamic spectra, and another custom-made ^13^C transceiver surface coil (3 cm in diameter) placed on the animal’s head for the brain EPSI data collection.

### ^1^H MRI

The ¹H MRI acquisition parameters are summarized in Table S2.

At 4.7 T, *T*_*1*_*-*weighted anatomical images of the kidneys were acquired using a Fast Low Angle Shot (FLASH) sequence with 25 contiguous slices of 1 mm each, TE = 1.7 ms, TR = 140 ms, flip angle = 50°, FOV = 75 × 55 mm^2^, and a matrix of 192 × 192. *T*_*2*_-weighted anatomical images of the liver were acquired using a Rapid Acquisition with Refocused Echoes (RARE) sequence with 5 contiguous slices of 2 mm each, TE = 45 ms, TR = 1600 ms, a RARE factor (i.e., echo train length) of 8, FOV = 60 × 40 mm^2^ and a matrix of 192 × 128.

At 9.4 T, a *T*_2_*-*weighted anatomical image of a 4-mm slice in the rat brain was acquired using a RARE sequence with TE = 33 ms, TR = 2500 ms, a RARE factor of 8, FOV = 80 × 32 mm^2^ and a matrix of 256 × 256.

### HP ^13^C MRI

The HP ^13^C acquisition parameters are summarized in Table S3.

At 4.7 T, the CSI of rat kidneys was acquired in a single slice 25 mm thick with TE = 1 ms, TR = 146 ms, flip angle = 10°, transmit bandwidth of 20,000 Hz, 1024 spectral points, 7142.9 Hz bandwidth, FOV = 75 × 55 mm^2^, matrix size = 15 × 11 (i.e., 5 mm in-plane resolution), 24.1 s temporal resolution, 8 repetitions, and a total scan time of 193 s. Data acquisition started 10 s after the start of injection of the hyperpolarized solution.

At 4.7 T, the EPSI experiment was performed in both healthy and tumor-bearing rats with a 10 mm axial slice over the liver using the following parameters: TE = 3 ms, TR = 255 ms, flip angle = 6°, EPSI gradient echo spacing = 0.2448 ms (i.e., 4085 Hz spectral bandwidth), 1024 spectral points, matrix size = 24 × 16 (phase encoding), FOV = 60 × 40 (phase encoding) mm² (i.e., 2.5 mm in-plane resolution), temporal resolution = 4.08 s, and 45 dynamic repetitions. Data acquisition started 8–9 s after the start of injection of the hyperpolarized solution.

AT 9.4 T, dynamic spectra were acquired over the rat kidney region with TR = 3 ms, flip angle = 10°, 1024 spectral points, and 80 dynamic repetitions. Data acquisition began at the start of injection of the hyperpolarized solution. The EPSI of the rat brain was acquired from a 4 mm slice using the following parameters: TE = 2.7 ms, TR = 255 ms, flip angle = 10°, EPSI gradient echo spacing = 0.1218 ms (i.e., 8210 Hz spectral bandwidth), 1024 spectral points, matrix size = 21 × 16 (phase encoding), FOV = 80 × 32 (phase encoding) mm² (i.e., 3.8 × 2 mm in-plane resolution), temporal resolution = 4.08 s, and 30 dynamic repetitions. Data acquisition began at the start of injection of the hyperpolarized solution.

### ^13^C spectral and image data processing

Spectra were reconstructed with custom-made MATLAB (Mathworks, MA) scripts, using line-broadening (50 Hz for CSI, 40 Hz for EPSI), zero-filling (2× spectral, 3× spatial), and processed with zero and first-order phasing, baseline correction, and peak integration. A step-by-step illustration of our post-processing method is described in our previous work^23^. Notably, baseline correction was performed effectively using an Asymmetric Reweighted Penalized Least Squares algorithm^39^ using a custom approach^23^. The ^13^C spectrum shown in Fig. 2B was derived from a hand-drawn ROI covering both the kidneys and subsequently summed over the first two frames. The ^13^C spectra in Fig. 3A, B were taken from the rectangular ROIs shown overlaid on the *T*_*2*_-weighted images and then summed over the first four frames of the acquisition. The magnitude spectra are shown in Fig. 3A, B. For the stack-plot in Fig. 4B, the ^13^C spectra were summed within a hand-drawn brain ROI and subsequently phased and baseline corrected.

## Supplementary information

Supplementary information

## Data Availability

All data generated and analyzed in this study are included in this published article and its Supplementary Information. Raw data are available from the corresponding author upon reasonable request.

## Code availability

The 13C MRSI data processing scripts in this work employ standard libraries and scripts that are available in MATLAB. A detailed description of the processing procedure is publicly available at https://pmc.ncbi.nlm.nih.gov/articles/PMC12273856/ and in its supplementary information https://pmc.ncbi.nlm.nih.gov/articles/instance/12273856/bin/NIHMS2095432-supplement-Supplementary_file.pdf.
