# Supplementary material for: Feasibility of co-polarizing ¹³C-pyruvate and ¹³C-tert-butanol for simultaneous metabolic and perfusion imaging
Source: Npj Imaging. 2026 May 18;4:43. doi: 10.1038/s44303-026-00172-9 (PMC13242510; doi:10.1038/s44303-026-00172-9)
Supplement: Supplementary file 1 — Supplementary information [file 44303_2026_172_MOESM1_ESM.pdf]

Title: Feasibility of Co-polarizing ^{13}C-Pyruvate and ^{13}C-Tert-Butanol for Simultaneous Metabolic and Perfusion Imaging

Supplementary information

Table S1: Liquid state polarization levels, relaxation time (T_1) and build-up times (T_p). *Liquid-state polarization (%) measured at 1.4 T and 27 °C and back-calculated to the moment of dissolution for all the sample formulations tested.*

	[1-^{13}C]pyruvate		^{13}C-TBA		
	Polarization %	T_1 s	Polarization %	T_1 s	T_p
12 mM AH [a]	25.2	75.3	-	-	1h 50 m
21 mM AH [a]	47.2	72.6	-	-	1h 07 m
25 mM AH [a]	46.9	68.4	-	-	47 m
25 mM AH [a]	44.9	62.5	-	-	59 m
30 mM AH [a]	34.3	66.1	-	-	31 m
37 mM AH [a]	38.0	67.2	-	-	33 m
45 mM AH [a]	30.5	64.7	-	-	28 m
21 mM OX [a]	35.9	72.2	-	-	1 h
25 mM OX [a]	32.4	76.8	-	-	31 m
30 mM OX [a]	33.2	66.0	-	-	35 m
21 mM AH [b]	42.6	64.5	40.2	29.4	1h 02 m
25 mM AH [b]	45.1	57.8	39.6	28.1	38 m
30 mM AH [b]	34.5	65.4	25.1	30.1	26 m
25 mM AH [c]	43.6	71.5	44.4	32.8	33 m

[a] Sample formulation using [1-^{13}C]pyruvate and natural abundance TBA. [b] Sample formulation using a mixture of equivolume of ^{13}C-labelled and natural abundance TBA. [c] Sample formulation using full volume of ^{13}C-labelled TBA.

The liquid state polarization using 25mM of Trityl AH was 45.1 ± 1.4 % (mean ± standard deviation) for [1-^{13}C]pyruvate (four samples) and 42.0 ± 3.4 % for ^{13}C-TBA (two samples).

Table S2: ^{1}H MRI Acquisition Parameters.

**Parameter**	**Kidneys (T1w)**	**Liver (T2w)**	**Brain (T2w)**
Field	4.7 T	4.7 T	9.4 T
Contrast	T_1-weighted	T_2-weighted	T_2-weighted
Sequence	FLASH	RARE	RARE
Number of slices	25 contiguous	5 contiguous	1
Slice thickness	1 mm	2 mm	4 mm
TE (ms)	1.7	45	33
TR (ms)	140	1600	2500
Flip angle (°)	50	–	–
RARE factor (echo train length)	–	8	8
Field of view	75 × 55 mm²	60 × 40 mm²	80 × 32 mm²
Matrix size	192 × 192	192 × 128	256 × 256

Table S3: HP ^{13}C MRI Acquisition Parameters

**Parameter**	**Kidneys (4.7 T, CSI)**	**Kidneys (9.4 T, Dynamic spectra)**	**Liver (4.7 T, EPSI)**	**Brain (9.4 T, EPSI)**
Coil	Bruker $^{1}H/^{13}C$ linear birdcage coil (72 mm inner diameter)	custom-built ^{13}C transceiver surface coil (6 cm in diameter)	custom-built ^{13}C transceiver surface coil (6 cm in diameter)	custom-built ^{13}C transceiver surface coil (3 cm in diameter)
Slice thickness	25 mm	–	10 mm	4 mm
TE (ms)	1	–	3	2.7
TR (ms)	146	3	255	255
Flip angle (°)	10	10	6	10
Echo spacing (ms)	–	–	0.2448	0.1218
Spectral bandwidth (Hz)	7142.9	–	4085	8210
Spectral points	1024	1024	1024	1024
Matrix	15 × 11	–	24 × 16	21 × 16
FOV (mm^2)	75 × 55	–	60 × 40	80 × 32
In-plane resolution	5 mm	–	2.5 mm	3.8 × 2 mm^2
Temporal resolution	24.1 s	–	4.08 s	4.08 s
Dynamic repetitions	8	80	45	30
Data acquisition	Started 10 s after injection	Started with injection	Started 8–9 s after injection	Started with injection
Total Scan	180 s	240 s	185 s	123 s

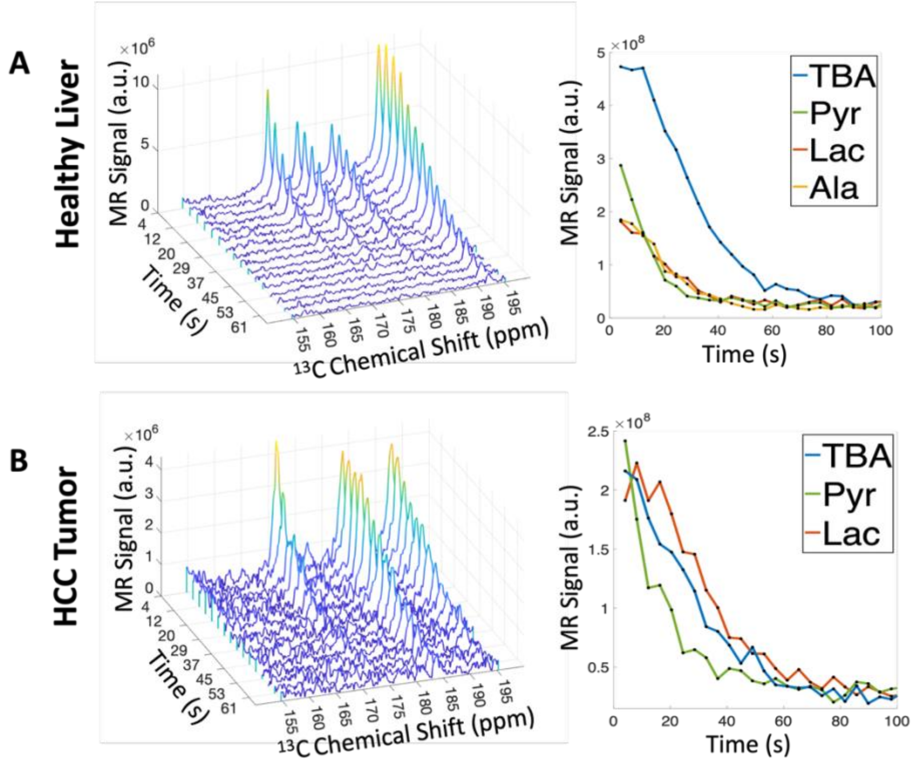

Figure S1: Temporal profiles of time-resolved EPSI of co-polarized [1-^{13}C]Pyruvate and ^{13}C-TBA in healthy (A) and tumor-bearing rat liver (B) at 4.7 T. *For each animal, temporal changes of the spectra summed over the liver or HCC tumor ROI (red boxes in Figure 2 in the main manuscript) are shown on the left and metabolite signal time curves shown on the right, illustrating the temporal evolution of the metabolite signals within the corresponding ROI.*

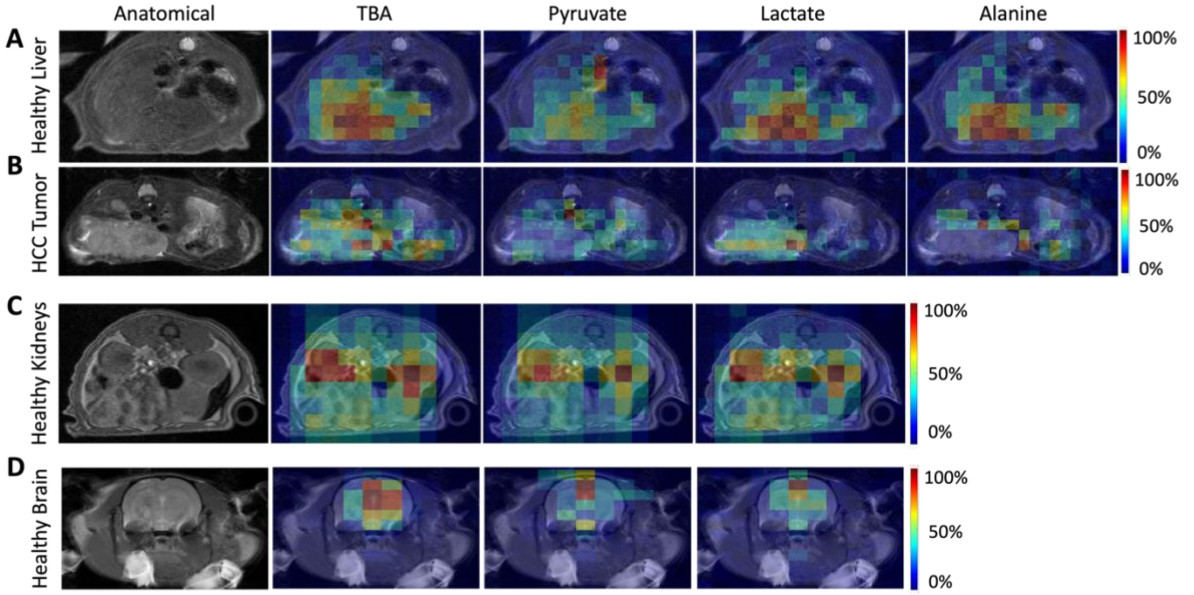

Figure S2: Non-interpolated metabolic images (i.e. no zero-fill in spectral nor spatial domains). *(A) EPSI of a rat with healthy liver at 4.7 T. (B) EPSI of a rat with HCC tumor at 4.7 T. (C) CSI of healthy rat kidneys at 4.7 T. (D) EPSI of a healthy rat brain at 9.4 T.*
